# Growth, chemical, and biochemical composition of *Conocarpus erectus* L. in response to NPK fertilizers and extracts of active dry yeast, seaweeds, and green tea

**DOI:** 10.3389/fpls.2025.1616150

**Published:** 2025-09-12

**Authors:** El-Sayed Mohamed El-Mahrouk, Huda Gamal Mahmoud, Khaled Abdelaal, Hayam Mohamed Aly Ebrahim, Ahmed Mohamed El-Tarawy, Waleed A. A. Alsakkaf, Temoor Ahmed, Hayssam M. Ali

**Affiliations:** ^1^ Horticulture Department, Faculty of Agriculture, Kafrelsheikh University, Kafrelsheikh, Egypt; ^2^ Agricultural Botany Department, Faculty of Agriculture, Kafrelsheikh University, Kafrelsheikh, Egypt; ^3^ Agricultural Research Center (ARC), Horticulture Research Institute (Antoniadis), Alexandria, Egypt; ^4^ Department of Botany and Microbiology, Collage of Science, King Saud University, Riyadh, Saudi Arabia; ^5^ Department of Plant Biotechnology, Korea University, Seoul, Republic of Korea

**Keywords:** *Conocarpus erectus*, chemical fertilizer, yeast, seaweeds, green tea

## Abstract

*Conocarpus erectus* L. is one of the ornamental shrubs or trees that are utilized in different aspects in landscape (gardens, cities, roads, etc.). Fertilization program is an essential factor affecting the aesthetic characters of this plant species. Pots experiment was conducted in a randomized completed split plot design with the aim to study the effects of NPK fertilizers at the levels of 100%, 75%, and 50% of the suggested doses of 18, 12, and 6 g/plant from ammonium sulfate, calcium superphosphate, and potassium sulfate, respectively, as well as natural extracts as active dry yeast (ADY) at 1 or 3 g L^-1^, green tea (GT) at 0.2 or 0.5 g L^-1^, and seaweeds (SW) at 1 or 1.5 mL L^-1^, and their interaction on the growth and attributes of *C. erectus* in the 2022 and 2023 seasons. The results indicated that 100% NPK dose produced the highest significant values of plant height, number of branches, primary stem diameter, area/leaf, chlorophyll index, shoots and root fresh and dry weight, percentage of relative water content (in the second season), N, P, K, and total carbohydrates in comparison with 75% and 50% NPK doses in the both seasons. Moreover, 100% NPK increased the activity of peroxidase enzyme, phenol compounds, and antioxidant activity over the 75% and 50% NPK doses. Conversely, 75% NPK recorded a high relative water content (in the first season) and activity of catalase and polyphenol oxidase enzymes over the 100% and 50% NPK doses. All treatments of natural extracts had positive impacts on the studied parameters previously mentioned when compared to untreated control. Among the natural extracts used, 3 g L^-1^ ADY was the best application to increase the studied traits, except for leaf area and P%, whose higher significant values resulted from treatments with application of SW extract at 1.5 mL L^-1^ and 1 g L^-1^ ADY + 0.2 g L^-1^ GT + 1 mL L^-1^ SW, respectively. Moreover, the 100% NPK dose combined with 3 gL^-1^ ADY was the best combination to execute the highest values of the aforementioned traits studied, except the primary stem diameter, P%, and PPO activity, whereas the highest values resulted from treatments with 100% NPK dose + 1.5 mL L^-1^ SW, 1 g L^-1^ ADY + 0.2 g L^-1^ GT + 1 mL L^-1^ SW, and 75% NPK dose + 3 g L^-1^ ADY, respectively. Therefore, 100% NPK treatment combined with 3 g L^-1^ADY is recommended to fertilize *C. erectus* plants in order to reduce the overuse of chemical fertilizers and to minimize habitat contamination for the maintenance of the whole environment.

## 1 Introduction

*Conocarpus erectus* L. is an evergreen tree or shrub (Combretaceae); it is native to Florida’s mangrove forest ecosystem in North America. It tolerates temperature up to 47 °C and grows in low-fertility soil (Nelson, 1996). It can resist high salinity levels if it is adequately watered. According to Popp et al. (1989), *C. erectus* provides food and cover for wildlife, helps fix dunes, and protects the soil during storm surges. It is commonly cultivated in various urban and landscaped settings such as gardens, parking areas, roadways, and public parks. Additionally, its potted forms are frequently utilized in the creation of bonsai (Gilman and Watson, 1993). Many factors affect the growth and chemical and biochemical processes in plant cells, consequently influencing the aesthetic value of ornamental plants. Significant changes to the morphological and physiological traits of plant parts (leaf, stem, and root) are identically attributed to plant growth, which is triggered with proper nutrition. Additionally, the soil physio-chemical characteristics can be altered directly or indirectly by fertilizer utilization (Li et al., 2020; Ren et al., 2020; Hou et al., 2023). Nutrition is an important factor among the factors that affect the growth and development of plants. Nitrogen (N), phosphorus (P), and potassium (K) are the major elements applied in fertilizing due to their essential roles in the metabolism of the plant cell, enzyme activity, and several processes of chemical, biochemical, and physiological aspects (Hawkesford et al., 2012). However, excessive application of traditional NPK can negatively affect the soil quality, eutrophicate bodies of water, and contaminate air and ground water (Congreves and Van Eerd, 2015). Moreover, the inefficient application of nutrients and habitat disruption pose significant challenges to sustainable agriculture and can result from the overuse of chemical fertilizers (Vanlauwe et al., 2010; Jones et al., 2013). To reduce the excessive use of chemical fertilizers, natural extracts can be utilized as substitutes or alternates.

Seaweed (SW) extract is standing as a novel agro-class of agro inputs originating from the horticultural world. Farmers either add seaweeds in the soil or apply it as a compost (Chojnacka et al., 2018). More recently, SW has been recognized as a biostimulant, defined as any substance or microorganism applied to plants with the aim of enhancing nutrient uptake, improving tolerance to abiotic stress, or increasing crop quality traits, irrespective of its inherent nutrient content. SW can be administered either through soil application or as a foliar spray (Du Jardin, 2015). The biochemical and chemical composition of SW is complex (polysaccharides, fats, proteins, oils, vitamins, elements, acids, hormones, pigments, and antioxidants (Khan et al., 2008; Craigie, 2011; Michalak and Chojnacka, 2014). According to Du Jardin (2015), seaweeds positively affect retention, remediation, and the microflora of soil, and they are considered a nutrient source; seaweeds have hormonal impacts as well. Seaweeds have also been proven useful owing to the actual content of molecule action in cell signaling, like polysaccharides (Bulgari et al., 2019; Franzoni et al., 2022), polyphenols, peptides, and carotenoids (Hrólfsdóttir et al., 2022), betaines, macronutrients, and micronutrients. Additionally, SW extracts contain some vital phytohormones such as auxins, gibberellins, and cytokinins, enhancing metabolism and development (Wang et al., 2016), in addition to other hormone-like substances (Petropoulos, 2020; Stirk et al., 2020). Therefore, seaweed application positively affects plant growth and development as shown in the studies of Kularathne et al. (2021) on ornamental plants, Mosa et al. (2021) on *Psidium juajava*, Radwan et al. (2023) on *Citrullus lanatus*, Loconsole et al. (2022) on *Lantana camara* and *Abelia grandiflora*, and Bagh et al. (2024) on strawberry.

Active dry yeast (ADY) (*Saccharomyces cerevisiae*) is considered a bio-stimulant (Hakobyan et al., 2012; Arastech Far et al., 2013). Yeast extract is a natural source of cytokinin and vitamin B (Matter and Abou-Sreea, 2016), protein, nucleic acids, most nutrient minerals, lipids, and carbohydrates. It also contains growth substances like pyridoxine, folic acid, vitamin B12, riboflavin, and thiamine (Nagodowithana, 1991). Moreover, the yeast cell wall contains polysaccharide substances (mannan B-glucan) that cause a major contribution to the antioxidant characteristic of yeast extract via their ability to scavenge hydroxyl free radical and superoxide anions (Liu et al., 2018). Especially the modification of B-glucan, by sulfation (Tang et al., 2017) or phosphorylation (Mei et al., 2019), can exhibit change in its physiochemical traits and biological activities, therefore further enhancing its antioxidant capacity. Furthermore, mannan has an amazing antioxidant characteristic (Ye et al., 2011). Abdelaal et al. (2021) documented that the utilization of yeast extract individually or combined with chitosan can improve the antioxidant enzyme’s activity and proline content but decreases oxidative stress such as what happened in garlic plants due to water stress. In addition, Alzandi and Naguib (2022) revealed that using yeast can increase the antioxidant enzyme’s activities and decrease lipid peroxidation.

The application of organic fertilizers on plants is recognized as an agricultural approach to stimulate the plants’ nutritional efficacy. As a result, implicating the use of organic fertilizers in plants and using them as a foliar spray have been the focus in order to facilitate its uptake and transmission into various plant organs (Niu et al., 2021; Vasundhara and Chhabra, 2021). Green tea (*Camellia sinensis*) is one of these plants’ organic fertilizers, and the essential role of applying green tea extract lies in its high and readily absorbable nutrient and vitamin levels, aside from the polyphenolic substances which act as antioxidants, as well as its role in boosting the activity of enzymes, leading to oxidative stress reduction (Armstrong et al., 2020). In addition, tannins, volatile oil, and caffeine are the vital compounds of green tea leaf extract (GTLE). In addition, GTLE contains catechin, epicatechin, epicatechin gallate, gallaocatechin, epigallaocatechin, and epigallaocatechin gallate, which are major active polyphenols (Nagle et al., 2006; Asadi et al., 2013).

Some studies focused on promoting the impact of GTLE on the growth and chemical and biochemical constituents of plants such as *Allium cepa* (Çavuşoğlu, 2020), *Aralia* plants (Moustafa, 2020), Barhee date plants (Abdel Aal et al., 2021), and *Ricinus communis* (Hassan and Ibrahim, 2024). Further research is necessary to gain a comprehensive understanding of the underlying mechanisms and to optimize the integration of yeast, seaweed, and green tea extracts with mineral nutrient applications. Currently, there is limited data available regarding the fertilization strategies for ornamental trees and shrubs. Therefore, this study focused on and suggested the response of *C. erectus* to various NPK doses, extracts of yeast, seaweeds, and green tea leaves and their interaction.

## 2 Materials and methods

At the experimental farm of the Faculty of Agriculture at Kafrelsheikh University in Egypt, pots experiment was conducted during the 2022 and 2023 seasons. The research aimed to study the response of vegetative growth, chemical and biochemical composition, enzymatic activities, and relative water content of *Conocarpus erectus* L. to NPK fertilizer doses, extracts (active dry yeast, seaweeds, and green tea), and their interaction. The farm’s location is in the coordinates 31°61′ N, 30°57′ E with an elevation of 6 m above sea level. The meteorological data recorded 203.28 and 111.68 mm of total rainfall, 53.68 and 57.73% average relative humidity, minimum average temperature of 15.0°C and 16.6°C, and average maximum temperature of 28.7°C and 29.6°C for the respective seasons according to the nearby Sakha meteorological station.

### 2.1 Plant material

Uniform *Conocarpus erectus* transplants, 3 months old and measuring 25 ± 2 cm in height with a stem diameter of 0.3 cm measured 3 cm above the soil surface, were obtained from the nursery of the Horticulture Department, Faculty of Agriculture, Kafrelsheikh University, for use in this study.

### 2.2 Soil analysis

The physical and chemical properties of the growth medium were analyzed prior to planting (**Table 1**). Following air-drying, the clay and sand mixture (1:1; v/v) was thoroughly homogenized and ground using a mortar and pestle. The processed sample was then sieved through a stainless steel mesh to obtain fractions smaller than 2 mm (Cools and De Vos, 2011). According to Gee and Bauder (1986), the hydrometer (US21CFR1040.10 AND 1040.11, USA) method was used to determine the distribution of the particle size.

**Table 1:** Physical and chemical parameters of the medium used.

Physical parameters		Chemical parameters			Available macronutrients (mg/kg)			Cations (meq/L)				Anions (meq/L)			
Clay	40.05	pH (1:2.5)	OM%	EC (Mmo-hs/ds)	N	P	K	Ca^++^	Mg^++^	Na^+^	K^+^	Cl^-^	CO_3_^–^	HCO_3_^-^	SO_4_^–^
Sand	37.03	7.84	1.33	1.53	1.56	2.27	163	3.3	3.1	6.93	0.17	1.73	–	8.5	3.27
Silt	22.92														
Soil texture	Clayey sand														

+cation monovalent; ++cation bivalent; -anion monovalent; –anions bivalent.

To evaluate the chemical characteristics of the medium, a 1:5 ratio was used by mixing 20 g of air-dried medium with 100 mL of distilled water. The suspension was allowed to stand for 24 h before being filtered. Electrical conductivity (EC) was then measured using an EC meter (MI170, SZ, Egged, Hungary, Italy) (Jackson, 1973). Magnesium (Mg^++^), calcium (Ca^++^), and chloride (Cl^-^) concentrations—ENREF-40—were estimated by using the methods of Jackson (1973). Standard methods were employed to determine the total carbonate content and organic matter (OM) of the medium (Nelson and Sommers, 1996). The available nitrogen (N) amount was measured with the micro Kjeldahl (DNB.1500 NPS.N.33848 made in Spain RAYPA) method (Bremner and Mulvaney, 1983). The available phosphorus (P) was estimated by using the procedure of Olsen and Sommers (1982). Sodium (Na^+^) and potassium (K^+^) were estimated using a PSC7 flame photometer (JENEWY, Staffordshire, UK) (Black et al., 1965). The pH of the medium was estimated in the suspension (1:2.5, medium: distilled water) after 30 min by using a pH meter (JENEWY3510, Staffordshire, UK) (Black et al., 1965).

### 2.3 Planting date

Comparable *C. erectus* transplants were planted individually in plastic pots (one transplant per pot) measuring 40 cm in diameter and 32 cm in height. Each pot was filled with 9 kg of the prepared growth medium, and planting was carried out in April 1 during both of the 2022 and 2023 growing seasons.

### 2.4 Experimental design

The experiment was designed as a randomized complete split plot design (Snedecor and Cochran, 1989), where NPK fertilizers doses were arranged as main plots and natural extracts were arranged as subplots. The experiment contained five repetitions, with each repetition including 15 treatments (three NPK doses × five natural extract levels). Each treatment of each repetition included two plants. It means that each treatment contained 10 plants for the five repetitions. Thus, the experiment included 150 plants in each season.

### 2.5 Fertilizers used

#### 2.5.1 Nitrogen, phosphorus, and potassium fertilizers

The N, P, and K at rates of 18, 12, and 6 g/pot as ammonium sulfate (20.5% N), calcium super phosphate (15.5% P_2_O_5_), and potassium sulfate (48% K_2_O) were applied as the suggested full dose. Calcium super phosphate was utilized as one utilization during medium preparation before planting. The rates of N and K fertilizers were divided into 6 equal doses and were added monthly starting in May 1 until October 1 of each season.

#### 2.5.2 Natural extracts

The natural extracts used were active dry yeast (ADY) at levels of 1 or 3 g L^-1^, seaweeds (SW) (*Spirulina platensis*, *Ecklonia maxima*) at levels of 1 or 1.5 mL L^-1^, and green tea (GT) (*Camellia sinensis*) at levels of 0.2 g L^-1^ or 0.5 g L^-1^. The ADY, SW, and GT extracts were sprayed three times with 1-month interval starting in May 2 during the experimental seasons. The plants were sprayed on the foliage until runoff in the morning. A wetting agent (Triton B) was added at 0.05 L^-1^ to all of the extracts used during spraying.

##### 2.5.2.1 Preparation of natural extracts

Active dry yeast extract was prepared by using nutritional media that contained glucose and casein as a suitable source of C and N at 6:1 ratio and other essential nutrients like K, P, Fe, Mn, and Mg, and the temperature of the incubation was set to match with that of Barnett et al. (1990). The yeast cell was activated under suitable aerobic and nutritional conditions to stimulate yeast vegetative production and the formation of beneficial bio-constituents (amino acids, auxins, cytokinins, vitamins, carbohydrates, protein, fatty acids, and enzymes) as well as the elements. The composition of ADY is presented in **Table 2**.

**Table 2:** Composition of the active dry yeast extract used.

Minerals (%)				Total protein (%)	Total carbohydrates (%)	Hormones (μg/mL)	
N	1.02	Zn	0.04	5.2	4.8	IAA	GA_s_
P	0.09	Cu	0.03			0.7	0.3
K	0.40	Fe	0.12				
Ca	0.03	Mn	0.05				
Na	0.008	Mo	0.0001				
Mg	0.01	B	0.013				

In the preparation of seaweed extract, 100 g of powder was soaked in 1 L of distilled water for 24 h using an orbital shaker to shake the extract at 25°C; then, the mixture was centrifuged (Sigma 3-18 KS, SIGMA Laborzentrifugen GMbH, Osterodeam Harz, Germany) at 5,000 rpm for 20 min to remove the debris. After that, Whatman no. 4 filter paper was used to filter the extract, and distilled water was added to complete the volume to 1 L (to obtain 10% w/v) of seaweed liquid extract. At 4°C, the extract was stored until use. The analysis of seaweed extract is presented in **Table 3**.

**Table 3:** Constituents of the seaweed extract used.

Macroelements (%)		Microelements (mg/L)		Hormones mg/L		Lactic acid (%)	Amino acids (%)	Matinol (%)
N	1	Fe	2,000	Cytokinins	10	5	2.2	10
P	5	Mn	1,000	IAA	200			
K	10	Zn	3,000	Gibberellins	0.04			

In the preparation of green tea extract, 20 g of dried GT leaves was placed in a 500-mL flask. At 25°C, 200 mL of distilled water was added. The sample was shaken at medium speed for 2 h. After that, the sample was left for an hour to settle. Afterward, it was filtered by using Whatman no. 4 filter paper to separate the residue from the filtrate. Then, 2 or 5 mL of the filtrate was completed to 1 L with the addition of distilled water to obtain the concentration used (0.2 or 0.5 g L^-1^) (Harborne, 1954).

### 2.6 Treatments applied

Main plots: NPK doses at 100%, 75%, and 50%.

Subplots: Natural extracts: control (distilled water), 3 g L^-1^ ADY, 0.5 g L^-1^ GT, 1.5 mL L ^-1^ SW, and 1 g L^-1^ ADY + 0.2 g L^-1^ GT + 1 mL L^-1^ SW.

### 2.7 Data recorded

After 7 months (in November 1 of each season) from the planting of *C. erectus* transplants, the measurements discussed in the following subsections were recorded.

#### 2.7.1 Vegetative traits

Plant height (cm), number of branches/plant, primary stem diameter (cm) at 5 cm above the soil surface, area/leaf (cm^2^) by a CI-202 laser area meter (CID Bio-Science, Cams, WA, USA), and chlorophyll index (SPAD units) were measured on the fifth leaf from the apical meristem using a portable leaf chlorophyll meter (SPAD-501; Minolta Crop., Osaka, Japan) (Markwell et al., 1995) as well as the fresh and dry weight of shoots (leaves + steams) and roots (g/plant). The plants were separated into shoots and roots. These parts were individually rinsed with tap water to remove the soil and further purified with distilled water. To determine the plant parts’ dry weight, they were dried in an oven at 80°C for 24 h (Rautio et al., 2010).

#### 2.7.2 Relative water content

The relative water content (RWC) percentage is as follows: five discs of *C. erectus* fresh leaves (1 cm diameter/disc) were weighed and then left to float on distilled water for 4 h to become fully turgid. The discs were weighed again (turgid weight). Afterward, the discs were dried at 70°C for 24 h and then weighed (dry weight) (Barrs, 1968). The RWC was calculated as follows:


$$RWC\;\%=\frac{\text{Fresh weight}-\text{Dry weight}}{\text{Turgid weight}-\text{Dry weight}}\times 100$$

#### 2.7.3 Assay of enzymatic activity

The enzymatic activity was determined for the second season only. Catalase (CAT) activity was determined using the method described by Aebi (1984), in which the decomposition of hydrogen peroxide (H_2_O_2_) was monitored by the decline in absorbance at 240 nm. The reaction mixture consisted of 20 mg of total protein, 10 mM H_2_O_2_, and sodium phosphate buffer (pH 7.0). CAT activity was expressed as the decrease in absorbance by 0.01 units at 240 nm per milligram of protein per minute.

Polyphenol oxidase (PPO) activity was estimated following the protocol of Malik and Singh (1980). The reaction mixture comprised 3.0 mL of 0.01 M buffered catechol and 0.1 M phosphate buffer (pH 6.0). Upon addition of 100 µL of crude enzyme extract, changes in absorbance at 495 nm were recorded every 30 s over a 3-min period. PPO activity was expressed as the increase in absorbance per minute per gram of fresh weight.

Peroxidase (POD) activity was assessed using the method of Hammerschmidt et al. (1982). The reaction mixture included 2.9 mL of 100 mM sodium phosphate buffer (pH 6.0), supplemented with 0.25% (v/v) guaiacol and 100 mM H_2_O_2_. Absorbance at 470 nm was measured at 30-s intervals for 3 min, and POD activity was expressed as the increase in absorbance per minute per gram of fresh weight.

#### 2.7.4 Total phenol compounds and antioxidant activity

Air-dried leaf samples were ground and extracted in methanol. After a 24-h soaking period, the mixture was filtered, and the total phenolic compounds (TPCs) in the filtrate were quantified. The concentration of TPCs in the crude extract was determined using an external calibration curve based on gallic acid and the Folin–Ciocalteu reagent as described by Singleton et al. (1999). Specifically, 0.2 mL of each extract was mixed with the Folin–Ciocalteu reagent. After 4 min, 1 mL of 15% sodium carbonate was added, and the mixture was left to stand at room temperature for 2 h. Absorbance was then measured at 760 nm using a spectrophotometer (Thermo Fisher Scientific, Waltham, MA, USA; model 4001/4). The TPC content was expressed as milligrams of gallic acid equivalents per gram of dry weight (mg GAE/g DW). Each value represented the average of five independent measurements per fraction (Martínez-Esplá et al., 2014).

The antioxidant activity (AOA) of the dry leaf extracts was assessed using a modified version of the 2,2-diphenyl-1-picrylhydrazyl (DPPH) radical scavenging assay, following the procedure of Binsan et al. (2008). A protein solution (0.1%) prepared in 5 mM phosphate-buffered saline (PBS, pH 7.2) was mixed with an equal volume (1:1 v/v) of 0.15 mM DPPH dissolved in 95% ethanol. The mixture was gently vortexed and incubated in the dark at room temperature for 30 min. The resulting absorbance was measured at 517 nm using a spectrophotometer (Helios Gamma; Thermo Fisher Scientific). A blank was prepared in the same manner, except that PBS was used in place of the sample. Trolox was employed to generate the calibration curve, with concentrations ranging from 12.5 to 100 µM. Antioxidant activity was expressed as Trolox equivalents (TE) per milligram of dry leaves. Both TPC and AOA measurements were conducted exclusively during the 2023 season.

#### 2.7.5 Leaf chemical composition

The digested leaves’ dry weight was prepared to estimate the percentages of N, P, K, and total carbohydrates. The leaf samples were dried for 24 h in the oven at 80°C. A homogeneous powder of leaves was prepared by grinding in a metal-free mill (Ika-Werke, M 20 Germany). Next, 0.2 g of the sample was mixed with 5 mL of concentrated sulfuric acid (95%), and the mixture was heated using a sand hotplate for 10 min. Then, 0.5 mL of perchloric acid was dropped carefully, and heating was continued to obtain a clear solution. The solution was filtered after cooling, and then it was diluted to 50 mL (Evenhuis and Dewaard, 1980). Micro Kjeldahl method was used to estimate N% (Chemists and Horwitz, 1990). A spectrophotometer (GT80^+^, UK) was used to quantify P% (Murphy and Riley, 1962). K% was measured by using an atomic absorption spectrophotometer (Cottenie et al., 1982). The techniques of Herbert et al. (1971) were utilized to estimate the total carbohydrates%.

### 2.8 Statistical analysis

Data analysis was performed using SAS software (version 6.12; SAS Institute Inc., Cary, NC, USA). Mean separation (± standard error) was conducted through a randomized complete split plot design, and significant differences among means were determined at *p ≤*0.05 using Duncan’s multiple-range test (DMRT).

## 3 Results

### 3.1 Vegetative growth traits

Vegetative traits in terms of plant height (PH), number of branches/plant (BN), primary stem diameter (PSD), leaf area (LA), chlorophyll index (CI), shoot fresh and dry weight (SFW, SDW), and root fresh and dry weight (RFW, RDW) were significantly affected by the application of different doses of NPK fertilizers, natural extracts, and their interaction in the two seasons (**Tables 4**–**6**).

**Table 4:** Effect of NPK doses, natural extracts, and their interaction on plant height, number of branches/plant, and primary stem diameter of *C. erectus* in the 2022 and 2023 seasons.

NPK doses (g/plant)	Natural extracts	Plant height (cm)		Branches number / plant		Primary stem diameter (cm)	
		1^st^ season	2^nd^ season	1^st^ season	2^nd^ season	1^st^ season	2^nd^ season
100% NPK		93.93 ±0.52 A	95.15 ±0.66 A	21.87 ±0.55 A	22.90 ±0.17 A	0.55 ±0.015	0.56 ±0.006 A
75 % NPK		76.13 ±0.13 B	77.20 ±0.46 B	19.47 ±0.73 B	20.40 ±0.35 B	0.47 ±0.024	0.50 ±0.016 B
50 % NPK		70.33 ±3.01 C	75.10 ±0.17 C	16.13 ±0.58 C	16.90 ±0.17 C	0.46±0.018	0.47 ±0.009 B
	Control	65.78 ±0.48 D	67.16 ±0.67 E	13.56 ±0.99 D	14.83 ±0.38 D	0.38 ±0.008 C	0.41±0.009 D
	3g L^-1^ ADY	96.61 ±0.58 A	99.08 ±0.43 A	23.00 ±0.69 A	23.50 ±0.48 A	0.53 ±0.020 A	0.56±0.024AB
	0.5g L^-1^ GT	70.67 ±2.0 C	72.83 ±0.29 D	16.22 ±0.33 C	17.50 ±0.38 C	0.44 ±0.005 B	0.45±0.005 C
	1.5ml L^-1^ SW	83.00 ±2.83 B	87.83 ±0.10 B	20.67 ±0.51 B	21.83 ±0.87 B	0.53 ±0.014 A	0.55±0.013 B
	1g L^-1^ ADY+0.2g L ^-1^ GT+ 1ml L^-1^ SW	84.61 ±0.71 B	85.50 ±0.29 C	22.33 ±0.33AB	22.66 ±0.19AB	0.57 ±0.014 A	0.58±0.011 A
100% NPK	Control	71.33 ±1.86 (e-h)	72.50 ±1.44 g	14.00 ±2.31 ef	16.00 ±1.15 gh	0.42 ±0.064 ef	0.47 ±0.035 e
	3g L^-1^ ADY	116.17 ±0.60 a	116.75 ±0.14 a	27.33 ±0.88 a	28.00 ±0.57 a	0.55 ±0.061abc	0.60 ±0.035 bc
	0.5g L^-1^ GT	80.00 ±2.89 de	82.50 ± 1.44 e	17.00 ±2.08 de	18.50 ±1.44 efg	0.45±0.019 cde	0.46 ±0.030 e
	1.5ml L^-1^ SW	99.67 ±2.73 b	101.50 ±2.02 b	25.00 ±1.00 a	25.50 ±0.87 ab	0.64 ±0.015 a	0.65 ±0.003 a
	1g L^-1^ ADY+0.2g L^-1^ GT+ 1ml L^-1^ SW	102.50 ±1.44 b	102.50 ±1.44 b	26.00 ±0.57 a	26.50 ±0.28 sb	0.64 ±0.006 a	0.64 ±0.006 ab
75 % NPK	Control	64.33 ±2.33 gh	66.50 ±0.87 ij	14.33 ±1.20 ef	15.50 ±0.29 hi	0.39 ±0.022 ef	0.41 ±0.003 f
	3g L^-1^ ADY	92.00±0.58 bc	92.50 ±0.29 c	24.67 ±0.67 ab	25.00 ±0.58 bc	0.53 ±0.058 bcd	0.57 ±0.032 c
	0.5g L^-1^ GT	65.33 ±0.88 gh	66.00 ±0.58 hi	17.00 ±1.15 de	18.00 ±0.58fgh	0.43 ±0.015 de	0.44 ±0.012 ef
	1.5ml L^-1^ SW	76.00 ±1.15 def	77.00 ±0.58 f	20.33 ±2.96 cd	22.50 ±2.02 cd	0.43 ±0.056 de	0.48 ±0.038 de
	1g L^-1^ ADY+0.2g L^-1^ GT+ 1ml L^-1^ SW	83.00 ±2.00 cd	84.00 ±1.73 e	21.00 ±1.00 bc	21.00 ±0.57de	0.56 ±a0.030 b	0.58 ±0.020 c
50% NPK	Control	61.67±0.88 h	62.50 ±0.29 j	12.33 ±0.90f	13.00 ±0.58 i	0.33 ±0.026 f	0.35 ±0.012 g
	3g L^-1^ ADY	81.67 ±1.76 cde	88.00 ±0.73 d	17.00 ±0.58 de	17.50 ±0.28 gh	0.52 ±0.007 bcd	0.52 ±0.006 d
	0.5g L^-1^ GT	66.67 ±3.33 fgh	70.00 ±0.00 gh	14.67 ±1.45 ef	16.00 ±0.57 gh	0.45 ±0.017 de	0.47 ±0.000 e
	1.5ml L^-1^ SW	73.33 ±12.02 (d-g)	85.00 ± 2.89 de	16.67 ±0.88 de	17.50 ±0.28 gh	0.52 ±0.000 bcd	0.52 ±0.006 d
	1g L^-1^ ADY+0.2g L^-1^ GT+ 1ml L^-1^ SW	68.33 ±1.76 fgh	70.00 ±0.57 gh	20.00 ±0.58 cd	20.50 ±0.29 def	0.52±0.007 bcd	0.52 ±0.006 d

Means that have the same letters within a column denote non-significance (*P* ≤ 0.05) according to Duncan’s multiple-range test (DMRT).

**Table 5:** Effect of NPK doses, natural extracts, and their interaction on leaf area (cm^2^) and shoots’ fresh and dry weight (g)/plant of *C. erectus* in the 2022 and 2023 seasons.

NPK doses (g/plant)	Natural extracts	Leaf area (cm^2^)		Shoots FW (g)/plant		Shoots DW (g)/plant	
		1st season	2nd season	1st season	2nd season	1st season	2nd season
100% NPK		60.32 ± 0.76 A	62.51 ± 1.27 A	831.01 ± 7.91 A	837.85 ± 3.93 A	207.73 ± 1.98 A	209.45 ± 0.98 A
75% NPK		55.89 ± 0.86 B	57.86 ± 1.14 B	798.10 ± 8.78 B	803.60 ± 5.60 B	195.51 ± 2.15 B	196.86 ± 1.37 B
50% NPK		43.84 ± 0.68 C	46.43 ± 1.88 C	673.14 ± 9.24 C	681.02 ± 4.62 C	161.52 ± 2.22 C	163.44 ± 1.11 C
	Control	47.52 ± 0.17 C	48.92 ± 0.81 D	695.07 ± 11.55 E	701.64 ± 7.79 E	170.63 ± 2.82 E	172.22 ± 1.91 E
	3 g L^-1^ ADY	58.27 ± 1.95 A	59.61 ± 1.41 A	846.81 ± 10.20 A	855.46 ± 5.10 A	207.58 ± 2.48 A	209.73 ± 1.24 A
	0.5 g L^-1^ GT	48.66 ± 3.30 BC	52.55 ± 2.25 C	716.77 ± 7.51 D	723.18 ± 3.75 D	194.47 ± 1.25 B	202.19 ± 0.61 B
	1.5 mL L^-1^ SW	58.85 ± 0.58 A	60.49 ± 1.10 A	758.47 ± 8.85 C	766.09 ± 4.43 C	186.05 ± 2.18 C	187.94 ± 1.09 C
	1 g L^-1^ ADY + 0.2 g L^-1^ GT + 1 mL L^-1^ SW	53.72 ± 1.50 AB	56.43 ± 1.57 B	819.95 ± 5.10 B	824.37 ± 2.50 B	182.55 ± 1.84 D	177.50 ± 0.92 D
100% NPK	Control	54.72 ± 1.91 bc	56.37 ± 0.96 e	780.11 ± 8.66 e	787.63 ± 4.33 h	195.00 ± 2.17 e	196.87 ± 1.08 f
	3 g L^-1^ ADY	70.74 ± 0.07 a	70.80 ± 0.03 a	885.17 ± 3.46 a	888.00 ± 1.73 a	221.25 ± 0.87 a	222.00 ± 0.43 a
	0.5 g L^-1^ GT	55.26 ± 5.95 bc	60.42 ± 2.97 cd	795.12 ± 9.81 d	803.53 ± 4.91 fg	198.75 ± 2.45 d	219.62 ± 0.51 b
	1.5 mL L^-1^ SW	61.69 ± 2.45 ab	63.81 ± 1.22 b	820.00 ± 13.28 c	831.50 ± 6.64 e	205.00 ± 3.32 c	207.87 ± 1.66 d
	1 g L^-1^ ADY + 0.2 g L^-1^ GT + 1 mL L^-1^ SW	59.18 ± 2.29 b	61.16 ± 1.14 bc	874.66 ± 4.33 a	878.60 ± 2.02 b	218.67 ± 1.08 a	200.87 ± 1.23 e
75% NPK	Control	55.56 ± 2.68 bc	57.88 ± 1.34 de	740.10 ± 12.12 g	740.25 ± 12.11 j	181.30 ± 2.97 h	181.30 ± 2.97 i
	3 g L^-1^ ADY	54.74 ± 0.76 bc	56.27 ± 0.88 e	855.15 ± 15.01 b	868.18 ± 7.51 c	209.48 ± 3.68 b	212.66 ± 1.84 c
	0.5 g L^-1^ GT	55.12 ± 1.78 bc	56.67 ± 0.89 e	755.06 ± 5.20 f	759.50 ± 2.60 i	208.25 ± 0.99 b	209.11 ± 0.50 d
	1.5 mL L^-1^ SW	57.08 ± 0.50 bc	57.51 ± 0.25 de	790.19 ± 7.51 de	796.55 ± 3.75 g	193.55 ± 1.84 ef	195.14 ± 0.92 fg
	1 g L^-1^ ADY + 0.2 g L^-1^ GT + 1 mL L^-1^ SW	56.93 ± 4.66 bc	60.97 ± 2.33 bc	850.00 ± 4.04 b	853.50 ± 2.02 d	184.98 ± 1.27 g	186.08 ± 0.64 h
50% NPK	Control	32.27 ± 0.26 e	32.50 ± 0.13 i	565.00 ± 13.86 j	577.17 ± 6.93 m	135.60 ± 3.33 l	138.48 ± 1.66 m
	3 g L^-1^ ADY	49.34 ± 4.12 cd	51.77 ± 3.33 f	800.12 ± 12.12 d	810.20 ± 6.06 f	192.00 ± 2.91 f	194.52 ± 1.45 g
	0.5 g L^-1^ GT	35.58 ± 5.75 e	40.56 ± 2.87 h	600.13 ± 7.51 i	606.50 ± 3.75 l	176.40 ± 1.66 i	177.84 ± 0.83 j
	1.5 mL L^-1^ SW	56.97 ± 6.68 bc	60.67 ± 1.84 cd	665.23 ± 5.77 h	670.23 ± 2.89 k	159.60 ± 1.39 j	160.80 ± 0.69 k
	1 g L^-1^ ADY + 0.2 g L^-1^ GT + 1 mL L^-1^ SW	45.04 ± 2.46 d	47.18 ± 1.23 g	735.20 ± 6.93 g	741.00 ± 3.46 j	144.00 ± 1.80 k	145.56 ± 0.90 l

Means that have similar letters in a column are non-significant (*P* ≤ 0.05) according to DMRT.

**Table 6:** Effect of NPK doses, natural extracts, and their interaction on chlorophyll index (SPAD units) and roots’ fresh and dry weight (g)/plant of *C. erectus* in the 2022 and 2023 seasons.

NPK doses (g/plant)	Natural extracts	Chlorophyll index (SPAD units)		Roots FW (g)/plant		Roots DW (g)/plant	
		1st season	2nd season	1st season	2nd season	1st season	2nd season
100% NPK		55.87 ± 1.36 A	57.78 ± 0.07 A	265.00 ± 14.32 A	277.40 ± 7.16 A	71.26 ± 4.97 A	75.50 ± 2.60 A
75% NPK		51.28 ± 0.89 B	51.45 ± 0.35 B	235.06 ± 11.09 B	244.90 ± 5.14 B	59.40 ± 3.29 B	62.00 ± 1.73 B
50% NPK		49.69 ± 0.62 C	51.25 ± 1.33 B	191.66 ± 10.34 C	200.70 ± 5.02 C	49.13 ± 2.49 C	51.20 ± 1.39 C
	Control	44.40 ± 1.25 E	45.18 ± 2.01 D	189.77 ± 9.43 E	198.00 ± 4.62 E	46.55 ± 3.76 E	49.67 ± 2.12 D
	3 g L^-1^ ADY	58.93 ± 1.96 A	61.83 ± 0.60 A	266.44 ± 12.51 A	277.33 ± 6.16 A	76.33 ± 3.73 A	79.33 ± 1.54 A
	0.5 g L^-1^ GT	47.63 ± 1.56 D	59.00 ± 0.27 B	205.22 ± 12.82 D	216.67 ± 5.77 D	53.55 ± 3.49 D	56.34 ± 2.11 C
	1.5 mL L^-1^ SW	52.11 ± 1.37 C	54.33 ± 0.26 C	241.33 ± 14.15 C	253.67 ± 6.93 C	59.44 ± 3.65 C	62.33 ± 1.73 B
	1 g L^-1^ ADY + 0.2 g L^-1^ GT + 1 mL L^-1^ SW	55.28 ± 1.84 B	56.28 ± 1.09 C	250.11 ± 10.68 B	259.33 ± 5.39 B	63.77 ± 3.66 B	66.83 ± 2.02 B
100% NPK	Control	47.66 ± 0.49 a	47.10 ± 0.23i	230.00 ± 12.70 f	241.00 ± 6.35 h	55.66 ± 7.51 fg	62.00 ± 4.04 ef
	3 g L^-1^ ADY	66.73 ± 3.70 a	69.65 ± 2.28 a	290.00 ± 13.86 a	302.00 ± 6.93 a	95.00 ± 4.04 a	98.50 ± 2.02 a
	0.5 g L^-1^ GT	51.06 ± 1.19 a	52.85 ± 0.89 g	250.00 ± 14.43 d	262.50 ± 7.22 e	60.66 ± 3.48 de	63.50 ± 2.02 e
	1.5 mL L^-1^ SW	58.43 ± 2.39 a	60.75 ± 0.61 c	275.00 ± 15.01 bc	288.00 ± 7.51 c	65.00 ± 4.62 d	69.00 ± 2.31 d
	1 g L^-1^ ADY + 0.2 g L^-1^ GT + 1 mL L^-1^ SW	55.46 ± 3.37 a	58.55 ± 1.36 d	280.00 ± 15.59 b	293.50 ± 7.79 b	80.00 ± 5.20 b	84.50 ± 2.60 b
75% NPK	Control	42.90 ± 2.27 a	44.50 ± 1.62 j	200.00 ± 7.51 i	206.50 ± 3.75 k	50.66 ± 2.33 h	52.50 ± 1.44 h
	3 g L^-1^ ADY	54.56 ± 4.53 a	58.90 ± 1.33 d	270.00 ± 10.39 c	279.00 ± 5.20 d	74.66 ± 4.33 c	78.50 ± 2.02 c
	0.5 g L^-1^ GT	47.33 ± 3.10 a	49.15 ± 2.51 h	211.00 ± 15.14 h	225.00 ± 7.55 i	56.33 ± 2.96 efg	58.50 ± 2.01 g
	1.5 mL L^-1^ SW	46.60 ± 2.90 a	49.40 ± 0.75 h	244.33 ± 13.85 de	256.50 ± 6.64 f	60.00 ± 3.79 e	62.50 ± 1.44 e
	1 g L^-1^ ADY + 0.2 g L^-1^ GT + 1 mL L^-1^ SW	55.93 ± 1.40 a	55.30 ± 0.29 f	250.00 ± 8.66 d	257.50 ± 4.33 f	55.33 ± 3.18 fg	58.00 ± 1.73 g
50% NPK	Control	42.63 ± 6.01 a	43.95 ± 4.19 g	139.33 ± 8.09 k	146.50 ± 3.74 m	33.33 ± 1.45 j	34.50 ± 0.87 c
	3 g L^-1^ ADY	55.50 ± 1.67 a	56.95 ± 0.84 e	239.33 ± 13.28 e	251.00 ± 6.35 g	59.33 ± 1.76 ef	61.00 ± 0.58 f
	0.5 g L^-1^ GT	44.50 ± 0.67 a	48.25 ± 0.14 hi	154.66 ± 8.95 j	162.50 ± 4.33 l	43.66 ± 4.06 i	47.00 ± 2.31 i
	1.5 mL L^-1^ SW	51.30 ± 1.68 a	52.85 ± 0.66 g	204.66 ± 13.57 hi	216.50 ± 6.64 j	53.33 ± 2.60 fg	52.50 ± 1.43 h
	1 g L^-1^ ADY + 0.2 g L^-1^ GT + 1 mL L^-1^ SW	54.50 ± 1.08 a	54.25 ± 0.83 hi	220.33 ± 7.80 g	227.00 ± 4.04 i	56.00 ± 2.65 fg	58.00 ± 1.73 g

Means that have the same letters in a column are non-significant (*P* ≤0.05) according to DMRT.

The 100% NPK dose significantly achieved the highest values of vegetative traits, followed by 75% NPK and ultimately 50% NPK. In the first and second seasons, the 100% NPK dose significantly resulted in the tallest PH (93.93 and 95.15 cm), the highest BN (21.87 and 22.90 branches/plant), the thickest PSD (0.53 and 0.54 cm), the largest LA (60.32 and 62.51 cm^2^/leaf), the highest CI (55.87 and 57.78 SPAD units), and the heaviest SFW (831.0 and 837.85 g/plant), SDW (207.73 and 209.45 g/plant), RFW (265.00 and 277.40 g/plant), and RDW (71.26 and 75.50 g/plant), respectively.

Conversely, all vegetative parameters were improved after spraying ADY, GT, and SW extracts individually or together relative to the untreated plants (control) during both experimental seasons (**Tables 4**-**6**). The significantly highest values of PH (96.61 and 99.08 cm), BN (23.00 and 23.50 branches/plant), CI (58.93 and 61.83 SPAD units), SFW (846.81 and 855.46 g/plant), SDW (207.58 and 209.73 g/plant), RFW (266.44 and 277.33 g/plant), and RDW (76.33 and 79.33 g/plant) had been recorded for the 3 g L^-1^ ADY treatment. In the same manner, the application of 1 g L^-1^ ADY + 0.2 g L^-1^ GT + 1 mL L^-1^ SW and 1.5 mL L^-1^ SW treatments resulted in high significant values of PSD (0.57 and 0.58 cm) and LA (58.85 and 60.49 cm^2^/leaf) in both seasons, respectively. The least significant values of PH (65.78 and 67.16 cm), BN (13.56 and 14.83 branches/plant), PSD (0.38 and 0.41 cm), LA (47.52 and 45.92 cm^2^/leaf), CI (44.40 and 45.18 SPAD units), SFW (695.07 and 701.64 g/plant), SDW (170.63 and 172.22 g/plant), RFW (189.77 and 198.00 g/plant), and RDW (46.55 and 49.67 g/plant) were recorded for unsprayed control plants in the two seasons, respectively. Meanwhile, the differences among the treatments of natural extracts reached a significant level (*P* ≤ 0.05) in most cases during both seasons. Additionally, the data in **Tables 4**-**6** indicate that 100% NPK combined with 3 g L^-1^ ADY led to the highest positively significant enhancement in vegetative traits, except for PSD whose higher values were recorded for the plants that received 100% NPK and sprayed with 1.5 mL L^-1^ SW and 1 g L^-1^ ADY + 0.2 g L^-1^ GT +1 mL L^-1^ SW in the two seasons. Conversely, the plants fertilized with 50% NPK without spraying of any extract resulted in the least significant values of vegetative traits in the both seasons. The other combinations between NPK doses and natural extracts recorded intermediate values of vegetative traits with significant differences among themselves in most cases during the two seasons.

### 3.2 Relative water content

It can be noted from the data in **Table 7** that the fertilized plants with either 100% NPK or 75% NPK had a higher RWC with the same level of significance (72.50% and 75.90% in the first season and 74.24% and 73.63% in the second one) in comparison with the fertilized plants by 50% NPK that had lower significance of relative water content (69.63% and 70.20%) in the two seasons. Moreover, the plants sprayed with 3 g L^-1^ ADY had the highest significant values of RWC (75.08% and 75.44%), while the untreated control plants had the least significant values of this item (66.98% and 68.05%) in both seasons. In the first season, the difference between ADY and GT or among GT, SW, and 1.0 g L^-1^ ADY + 0.2 g L^-1^ GT + 1 mL L^-1^ SW treatments did not reach a significant level (*P* ≤ 0.05). This fact is between SW and 1 g L^-1^ ADY + 0.2 g L^-1^ GT + 1.0 mL L^-1^ SW treatments in the second season. Furthermore, the combined applications of 100% NPK with 3 g L^-1^ ADY or 0.5 g L^-1^ GT extracts caused a higher significance of RWC at 76.87% and 75.38% in the first season and 77.16% and 77.50% in the second one, respectively. On the contrary, the plants that received 50% NPK without treating their leaves with natural extract had a lower significant RWC of 60.79% and 62.34% for the two seasons, respectively. The RWC indicated that the differences among the most combinations of NPK doses and natural extracts used did not reach a significant level in both seasons.

**Table 7:** Effect of NPK doses, natural extracts, and their interaction on relative water content% and total carbohydrates% of *C. erectus* in the 2022 and 2023 seasons.

NPK doses (g/plant)	Natural extracts	RWC%		Total carbohydrates%	
		1st season	2nd Season	1st season	2nd season
100% NPK		72.50 ± 0.35 A	74.24 ± 0.02 A	23.94 ± 0.44 A	25.52 ± 0.27 A
75% NPK		72.90 ± 0.08 A	73.63 ± 0.35 A	22.70 ± 0.14 B	23.44 ± 0.08 B
50% NPK		69.63 ± 0.92 B	70.20 ± 0.39 B	22.06 ± 0.38 B	21.93 ± 0.19 C
	Control	66.98 ± 0.52 C	68.05 ± 0.14 D	16.36 ± 0.05 E	17.08 ± 0.17 D
	3 g L^-1^ ADY	75.08 ± 0.13 A	75.44 ± 0.11 A	27.64 ± 0.07 A	27.87 ± 0.03 A
	0.5 g L^-1^ GT	72.91 ± 0.91 AB	74.31 ± 0.01 B	20.17 ± 0.14 D	21.35 ± 0.07 B
	1.5 mL L^-1^ SW	71.94 ± 1.01 B	73.07 ± 0.20 C	23.44 ± 0.04 C	24.13 ± 0.01 C
	1 g L^-1^ ADY + 0.2 g L^-1^ GT + 1 mL L^-1^ SW	71.68 ± 0.93 B	72.60 ± 0.08 C	26.90 ± 0.26 B	27.72 ± 0.21 A
100% NPK	Control	71.62 ± 1.75 c-f	72.86 ± 1.23 def	17.66 ± 0.66a	18.54 ± 0.55a
	3 g L^-1^ ADY	76.87 ± 0.41 a	77.16 ± 0.29 cde	30.12 ± 0.84a	31.84 ± 0.42a
	0.5 g L^-1^ GT	75.38 ± 2.13 abc	77.50 ± 0.15 a	22.15 ± 0.56a	22.63 ± 0.28a
	1.5 mL L^-1^ SW	68.26 ± 2.24 ef	70.41 ± 0.65 gh	25.45 ± 1.18a	26.47 ± 0.61a
	1 g L^-1^ ADY + 0.2 g L^-1^ GT + 1 mL L^-1^ SW	73.08 ± 0.33 a-d	73.29 ± 0.25 c-f	28.74 ± 0.67a	28.12 ± 0.55a
75% NPK	Control	67.93 ± 1.19 f	68.95 ± 0.60 h	16.22 ± 1.10a	17.17 ± 0.55a
	3 g L^-1^ ADY	74.58 ± 0.32 abc	74.82 ± 0.20 bc	27.14 ± 1.38a	26.33 ± 0.69a
	0.5 g L^-1^ GT	73.10 ± 0.98 a-d	73.80 ± 0.69 cde	21.32 ± 0.56a	21.81 ± 0.28a
	1.5 mL L^-1^ SW	75.50 ± 0.90 ab	76.12 ± 0.65 ab	23.14 ± 0.90a	23.93 ± 0.45a
	1 g L^-1^ ADY + 0.2 g L^-1^ GT + 1 mL L^-1^ SW	73.39 ± 1.35 a-d	74.48 ± 0.80 bcd	25.30 ± 0.77a	27.95 ± 0.42a
50% NPK	Control	60.79 ± 1.57 g	62.34 ± 0.22 i	15.02 ± 0.59a	15.53 ± 0.30a
	3 g L^-1^ ADY	73.80 ± 0.60 a-d	74.35 ± 0.25 a	24.80 ± 0.75a	25.45 ± 0.38a
	0.5 g L^-1^ GT	70.26 ± 1.48 def	71.63 ± 0.55 fg	19.00 ± 0.69a	19.60 ± 0.35a
	1.5 mL L^-1^ SW	72.06 ± 0.86 b-e	72.68 ± 0.60 ef	21.64 ± 0.40a	21.99 ± 0.20a
	1 g L^-1^ ADY + 0.2 g L^-1^ GT + 1 mL L^-1^ SW	68.56 ± 1.50 ef	70.03 ± 0.32 gh	26.51 ± 0.66a	27.09 ± 0.32a

Means that have identical letters within a column are non-significant (*P* ≤ 0.05) according to DMRT.

### 3.3 Leaf nitrogen, phosphorus, potassium, and total carbohydrate percentages

The data on total carbohydrates (TC) (**Table 7**), N%, P%, and K% (**Table 8**) show that the percentages of these traits were differently impacted in response to the NPK levels used in the two seasons. Moreover, the 100% NPK resulted in higher percentages of N, P, K, and total carbohydrate, recorded as 2.94%, 0.56%, 3.62%, and 23.94% in the first season and 2.98%, 0.61%, 3.65%, and 25.52% in the second season, respectively. Conversely, the 50% NPK dose suggested lower significant N, P, K and TC% values in the first season, resulting in 2.82%, 0.40%, 3.07%, and 22.08%, and in the second one recorded as 2.88%, 0.42%, 2.54%, and 21.93%, respectively.

**Table 8:** Effect of NPK doses, natural extracts, and their interaction on nitrogen (%), phosphorus (%), and potassium (%) of *C. erectus* in the 2022 and 2023 seasons.

NPK doses (g/plant)	Natural extracts	N (%)		P (%)		K (%)	
		1st season	2nd season	1st season	2nd season	1st season	2nd season
100% NPK		2.94 ± 0.03A	2.98 ± 0.03 A	0.56 ± 0.01 A	0.61 ± 0.02A	3.62 ± 0.12 A	3.65 ± 0.12 A
75% NPK		2.89 ± 0.03A	2.89 ± 0.02 B	0.47 ± 0.01B	0.51 ± 0.02 B	3.27 ± 0.11 B	3.37 ± 0.13 B
50% NPK		2.82 ± 0.03A	2.88 ± 0.02 B	0.40 ± 0.01 C	0.42 ± 0.00 C	3.07 ± 0.03 C	2.54 ± 0.09 C
	Control	2.62 ± 0.01 B	2.71 ± 0.02 C	0.38 ± 0.03 B	0.41 ± 0.03 C	2.60 ± 0.04 D	2.75 ± 0.04 D
	3 g L^-1^ ADY	2.98 ± 0.05 A	3.00 ± 0.05 A	0.46 ± 0.00 B	0.50 ± 0.02 B	4.12 ± 0.03 A	4.15 ± 0.03 A
	0.5 g L^-1^ GT	2.91 ± 0.01 A	2.93 ± 0.01 B	0.45 ± 0.01 B	0.47 ± 0.01 BC	3.56 ± 0.02 B	3.06 ± 0.19 C
	1.5 mL L^-1^ SW	2.96 ± 0.03 A	2.99 ± 0.02 A	0.47 ± 0.00 B	0.53 ± 0.01 B	3.04 ± 0.02 C	3.09 ± 0.03 C
	1 g L^-1^ ADY + 0.2 g L^-1^ GT + 1 mL L^-1^ SW	2.94 ± 0.03 A	2.96 ± 0.02 AB	0.64 ± 0.00 A	0.68 ± 0.01 A	3.79 ± 0.02 B	3.85 ± 0.02 B
100% NPK	Control	2.60 ± 0.23 ef	2.70 ± 0.12 hi	0.50 ± 0.01 b–e	0.33 ± 0.04 h	2.81 ± 0.07 e	2.85 ± 0.03 d
	3 g L^-1^ ADY	3.10 ± 0.03 a–d	3.15 ± 0.01 cd	0.51 ± 0.07 b–e	0.47 ± 0.00 ef	4.60 ± 0.06 a	4.60 ± 0.06 a
	0.5 g L^-1^ GT	2.95 ± 0.03 a–d	2.97 ± 0.01 cd	0.55 ± 0.05 bcd	0.37 ± 0.01 gh	3.08 ± 0.04 e	3.08 ± 0.04 d
	1.5 mL L^-1^ SW	3.05 ± 0.03 ab	3.07 ± 0.01 ab	0.64 ± 0.14 ab	0.40 ± 0.00 gh	3.09 ± 0.04 e	3.13 ± 0.01 d
	1 g L^-1^ ADY + 0.2 g L^-1^ GT + 1 mL L^-1^ SW	2.98 ± 0.06 abc	2.99 ± 0.03 cd	0.61 ± 0.06 abc	0.56 ± 0.02 cd	4.51 ± 0.09 a	4.58 ± 0.04 a
75% NPK	Control	2.75 ± 0.03 de	2.77 ± 0.01 gh	0.35 ± 0.03 ef	0.38 ± 0.01 gh	2.74 ± 0.62 ef	3.08 ± 0.52 d
	3 g L^-1^ ADY	2.88 ± 0.01 bcd	2.89 ± 0.01 ef	0.42 ± 0.07 def	0.47 ± 0.03 ef	4.16 ± 0.07 ab	4.21 ± 0.04 b
	0.5 g L^-1^ GT	2.83 ± 0.01 cd	2.84 ± 0.01 fg	0.42 ± 0.01 def	0.43 ± 0.00 fg	3.18 ± 0.03 de	3.18 ± 0.03 d
	1.5 mL L^-1^ SW	3.00 ± 0.06 abc	3.05 ± 0.03 bc	0.41 ± 0.03 def	0.43 ± 0.02 efg	3.14 ± 0.05 de	3.21 ± 0.01 d
	1 g L^-1^ ADY + 0.2 g L^-1^ GT + 1 mL L^-1^ SW	2.88 ± 0.04 bcd	2.91 ± 0.02 def	0.77 ± 0.06 a	0.81 ± 0.04 a	3.14 ± 0.04 de	3.16 ± 0.02 c
50% NPK	Control	2.50 ± 0.01 f	2.65 ± 0.01 i	0.29 ± 0.05 f	0.51 ± 0.01 de	2.26 ± 0.05 f	2.33 ± 0.03 e
	3 g L^-1^ ADY	2.95 ± 0.06 a	2.97 ± 0.03 a	0.47 ± 0.00 cde	0.57 ± 0.04 cd	3.59 ± 0.07 cd	3.64 ± 0.04 c
	0.5 g L^-1^ GT	2.95 ± 0.03 a–d	2.97 ± 0.01 cd	0.36 ± 0.02 e	0.60 ± 0.02 bc	2.91 ± 0.02 e	2.93 ± 0.01 d
	1.5 mL L^-1^ SW	2.83 ± 0.01 cd	2.84 ± 0.01 fg	0.36 ± 0.02 ef	0.75 ± 0.07 a	2.90 ± 0.04 e	2.93 ± 0.03 d
	1 g L^-1^ ADY + 0.2 g L^-1^ GT + 1 mL L^-1^ SW	2.95 ± 0.03 a–d	2.96 ± 0.02 cd	0.53 ± 0.03 bcd	0.66 ± 0.04 b	3.72 ± 0.70 bc	3.80 ± 0.03 c

Means that have the same letters within a column are non-significant (*P* ≤ 0.05) according to DMRT.

The natural extracts used caused positive effects in increasing N, P, K, and TC% over the untreated control treatment. Furthermore, spraying 3 g L^-1^ ADY gave higher N (2.98% and 3.80%), K (4.12% and 4.15%), and TC (27.64% and 27.87%) in the two seasons, respectively. The plant leaves treated with 1 g L^-1^ ADY + 0.2 g L^-1^ GT + 1 mL L^-1^ SW contained higher percentages of P (0.64% and 0.68%) for both seasons. Regardless of the control, the differences among the treatments of natural extracts reached a significant level in some cases, especially in the case of TC in the two seasons.

Concerning the interaction impact of NPK doses and natural extracts, the data in **Tables 7** and **8** demonstrate that the leaves of plants fertilized with 100% NPK and sprayed with 3 gL^-1^ ADY contained higher TC% (30.12% and 31.84%), N% (3.10% and 3.15%), and K% (4.60% and 4.60%) in the both seasons, respectively. The leaves of plants that received 75% NPK and were sprayed with 1 g L^-1^ ADY + 0.2 g L^-1^ GT + 1 mL L^-1^ SW contained higher P% (0.77% and 0.81%) in the two seasons. Conversely, lower significant percentages of N (2.50% and 2.65%), P (0.29% and 0.33%), K (2.26% and 2.33%), and TC (15.02% and 15.53%) were found in the leaves of plants fertilized with 50% NPK and not sprayed with any natural extract used in the two seasons.

### 3.4 Antioxidant enzyme activity

**Figure 1:**
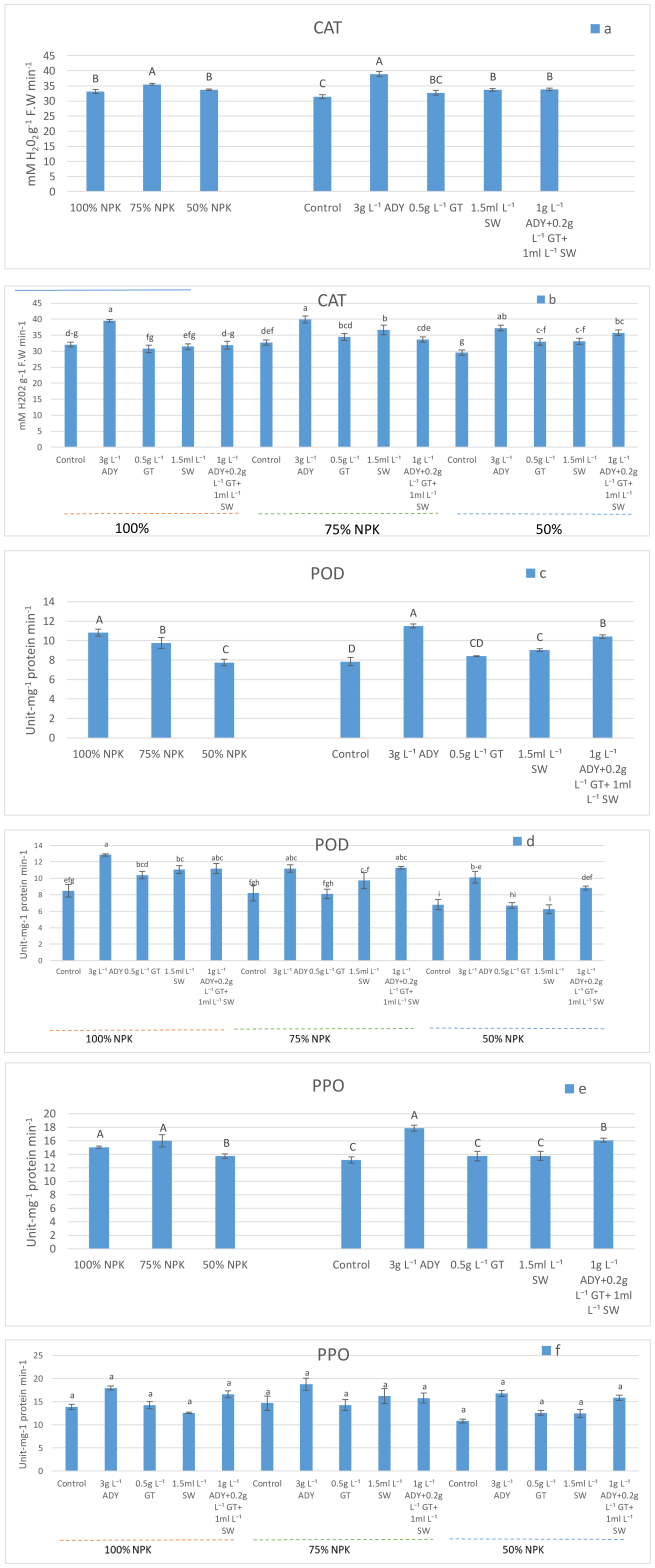
Effect of NPK doses, natural extracts, and their interaction on the activities of CAT **(a, b)**, POD **(c, d)**, and PPO **(e, f)**. Within a figure, means that have similar letters denote non-significance (*P* ≤ 0.05) according to DMRT.

The activities of CAT, POD, and PPO enzymes were significantly affected by the application of NPK doses. The 75% NPK caused a higher significant CAT activity (35.48 mM H_2_O_2_ g^-1^ FW min^-1^) in comparison with 100% and 50% NPK which recorded 33.13 and 33.74 mM H_2_O_2_ g^-1^ FW min^-1^, respectively (**Figure 1a**), with the same significance level. Meanwhile, the leaves of fertilized plants with 100% NPK had the highest significant POD activity (10.80 unit-mg^-1^ protein min^-1^, followed by 75% NPK (9.76 unit-mg^-1^ protein min^-1^), and ultimately 50% NPK (7.74 unit-mg^-1^ protein min^-1^) (**Figure 1c**). At the same time, 100% and 75% caused a higher significant PPO activity (15.06 and 15.97 unit-mg^-1^ protein min^-1^, respectively) with the same significance level. In the second rank lies 50% NPK which enriched the PPO activity to 13.71 unit-mg^-1^ protein min^-1^ (**Figure 1e**). All treatments of natural extracts increased the activities of CAT (**Figure 1a**), POD (**Figure 1c**), and PPO (**Figure 1e**) more than the control treatment. Moreover, the leaves of plants sprayed with 3 g L^-1^ ADY had the largest significant activities of such enzymes when compared to the other natural extracts used. Under the application of 3 g L^-1^ ADY, the CAT activity reached 38.89 mM H_2_O_2_ g^-1^ FW min^-1^, and the POD and PPO activities reached 11.5 and 17.85 unit-mg^-1^ protein min^-1^, respectively. Meanwhile, a lower significant activity was found in the leaves of control plants at 31.44 mM H_2_O_2_ g^-1^ FW min^-1^ for CAT and 7.83 and 13.14 unit-mg^-1^ protein min^-1^ for POD, and PPO, respectively.

Meantime, the interaction of combinations between NPK doses and natural extracts exhibited various effects on CAT, POD, and PPO activities. The combined 3 g L^-1^ ADY with 100%, 75%, and 50% NPK levels caused a significantly higher CAT activity (**Figure 1b**) reaching 39.50, 39.90, and 37.26 mM H_2_O_2_ g^-1^FW min^-1^, respectively, which took place at the level of significance. On the other hand, the combinations of 3 g L^-1^ ADY and 1 g L^-1^ ADY + 0.2 g L^-1^ GT + 1 mL L^-1^ SW with either 100% NPK or 75% NPK doses were the best for activating POD enzyme, reaching 12.86, 11.20, 11.20, and 11.26 unit-mg^-1^ protein min^-1^, respectively (**Figure 1d**). However, the activity of PPO enzyme reached 18.80 unit-mg^-1^ protein min^-1^ after utilizing 3 g L^-1^ ADY with 75% NPK (**Figure 1f**). Conversely, the least activities of CAT, POD, and PPO were noticed in the levels of plants fertilized with 50% NPK without spraying with any natural extracts, containing 29.56 mM H_2_O_2_ g^-1^ FW min^-1^, and 6.23 and 10.83 unit-mg^-1^ protein min^-1^, respectively. Additionally, the impacts of the various combinations of NPK doses and natural extracts on enzyme activity were significant in some cases for either CAT or POD but not significant in the case of PPO (*P* ≤ 0.05).

### 3.5 Leaf total phenol compounds and antioxidant activity

**Figure 2:**
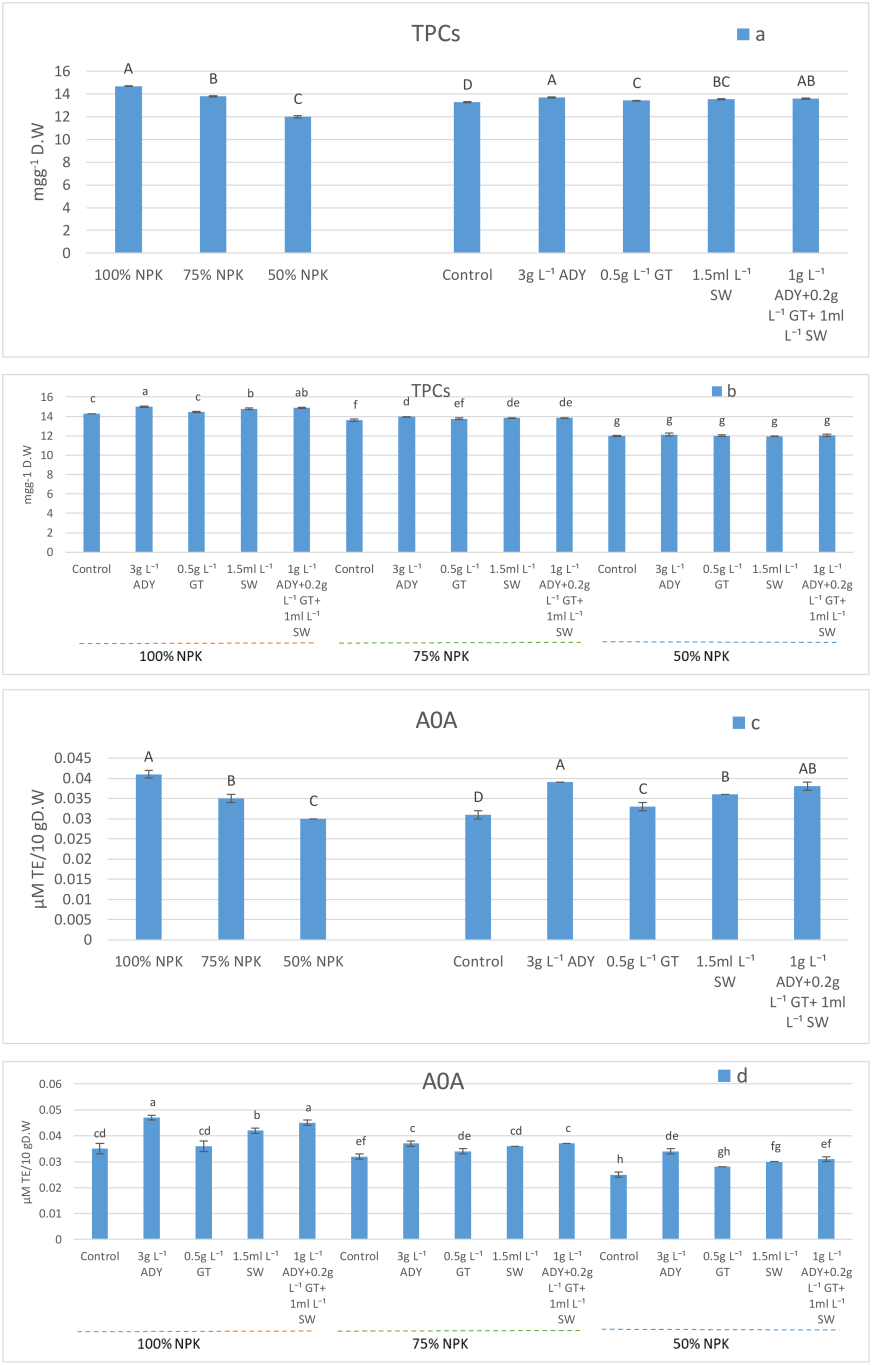
Effect of NPK doses, natural extracts, and their interaction on the content of TPCs **(a, b)** and AOA **(c, d)**. Means that have the same letters are non-significantly different (*P* ≤ 0.05) according to DMRT.

Total phenol compounds (TPCs) and antioxidant activity (AOA) were significantly affected by the application of various NPK doses (**Figures 2a, c**), whereas 100% NPK was the best in enhancing TPCs and AOA at 14.69 mg/g DW and 0.04 μM TE/10 g DW, respectively. Moreover, as the NPK level decreased, the TPCs and AOA decreased. On the other hand, such parameters showed significant differences under various natural extracts used (**Figures 2a, c**). The use of 3 g L^-1^ ADY and 1 g L^-1^ ADY + 0.2 g L^-1^ GT + 1 mL L^-1^ SW treatments resulted in higher significant values of TPCs (13.69 and 13.59 mg/g DW) and AOA (0.039 and 0.038 μM TE/10 g DW), respectively, with the same significance level (*P* ≤ 0.05). It is noticed that all of the natural extracts used significantly improved such two parameters relative to the control treatment which gave lower significant values (13.28 mg/g DW and 0.031 μM TE/10 g DW, respectively). In regard to the NPK levels and natural extracts used, the interaction data listed in **Figures 2b, d** show that the leaves of plants fertilized with 100% NPK and sprayed with either 3 g L^-1^ ADY or 1 g L^-1^ AD + 0.2 g L^-1^ GT + 1 mL L^-1^ SW had the highest significant values of TPCs (15.01 and 14.88 mg/g DW) and AOA (0.047 and 0.045 μM TE/10 g DW), respectively, and the two treatments took place at the same significance level (*P* ≤ 0.05). Conversely, the plants fertilized with 50% NPK without receiving any natural extract showed that their leaves have lower significant values of TPCs (11.96 mg/g DW) and AOA (0.025 μM TE/10 g DW). The differences among the combinations of NPK doses and natural extracts used did not reach a significant level in most cases.

## 4 Discussion

### 4.1 Impact of N, P, K fertilizers on vegetative, chemical, and biochemical traits

N, P, and K are the major nutrients for high-quality plant traits, and proper utilization can positively enhance plant growth and structure (Nemadodzi et al., 2017; Xing et al., 2023). A close relationship exists among nutrients, soil, and plants, whereby soil fertility—which influences plant traits—is, in turn, affected by the appropriateness of the fertilizers used (Chen et al., 2019; Zhang et al., 2023). Our study showed that 100% NPK dose was the most effective rate in increasing the vegetative traits and chemical and biochemical parameters except for RWC (in the first season) and the activity of CAT and PPO where 75% NPK was optimal. NPK fertilizers also have essential roles in plant physiological processes such as in enhancing enzyme activities, in the translocation of carbohydrates as a product of photosynthesis process, and in cell division and elongation, which essentially affect a plant’s nutritional status. NPK application leads to an increase in IAA level and photosynthesis process efficiency, thus raising carbohydrate production (Alkhafaji and Khalil, 2019), which improves the traits being studied. Additionally, NPK have important roles in protoplasm formation and element biosynthesis for several compounds within plant cells, like amino acids, nucleic acids, amides, proteins, building chlorophyll, activation of some enzymes, meristematic growth, and energy-carrying formation (Al-Marsoumi et al., 2020; Al-Khafaji et al., 2022). Choudhary et al. (2022) concluded as well that proper NPK availability is best matched to a higher N uptake which, in turn, improves the quantity of mesophyll cells and chloroplasts and chlorophyll stability, raising the content of chlorophyll in plants. Relevant research have clarified that suitable fertilization of N, P, and K can effectively raise the leaves’ and soil’s nutrient content, thereby boosting plant yield and quality (Zhang et al., 2020; Wan et al., 2021; Zhang et al., 2022). The N, P, and K proportions are often unbalanced owing to the scientific ratio lacking in actual production, which lead to the plant absorption and nutrient application being affected, a decrease in yield and quality, and increased risk of nutrient loss and habitat contamination. Thus, the N, P, and K appropriate ratio can positively enhance plant growth and decrease the usage of various fertilizers relatively (Yang et al., 2020). According to Alhasan et al. (2022), 75 kg N/ha as urea significantly improved chamomile growth and flowering. In addition, Shi et al. (2021) reported that biomass production of lettuce can be maximized by the proper management of fertilizer. Conversely, the overuse application of fertilizer can cause disease episodes which can negatively affect leaf and root development and nitrate accumulation. Rose leaf dry weight and chlorophyll content were significantly increased by NPK application at 5 g L^-1^ (Jawad and Khalil, 2024). Our results confirm the findings of Liu et al. (2024) who found that different combinations of N, P, and K significantly increased the leaf area index, chlorophyll, soluble sugar, starch, and N, P, and K contents of *Sapindus mukorossi* leaves. Also, Nofal et al. (2021) concluded that 2 g/pot applied three times with a 15-day interval positively improved chlorophyll a and b, carotenoids, percentages of total carbohydrates, N, P, K, total indoles, and total phenols in the leaves of *Tagetes erecta*. Related to this concern, Choudhary et al. (2022), in their study on *Chrysanthemum morifolium*, found that significant increases in plant height, number of branches/plant, leaf area, fresh and dry weight, flowering span, total fresh and dry weight of flowers, and chlorophyll content values were gained up to 300 kg N/ha. The tallest plants, the biggest number of branches, and the highest chlorophyll index of *Turnera ulmifolia* were recorded under the application of NPK (15:15:15) at 0.2 g/plant for three time with a 1-month interval (Sidi et al., 2022). As clarified by Jiang et al. (2024), the combination of N, P_2_O_5_, and K_2_O at 330, 196, and 450 kg/ha, respectively, significantly produced the highest values of plant height, stem diameter, and leaf area in the seedling stage of *Chrysanthemum morifolium*, while the highest significant values of such traits and chlorophyll content resulted from N, P_2_O_5_, and K_2_O at 330, 196, and 300 kg/ha, respectively, during the branching and flowering stages. Indeed to avoid the harmful impact of reactive oxygen species (ROS), the plants should have an effective antioxidant system. The defense systems produce enzymes which help repair the toxicity brought about by ROS and enhance the stability of plants to resist diseases. PPO, POD, CAT, superoxide dismutase (SOD), and phenylalanine ammonia lyase (PAL) act as defense-related enzymes that have an essential role on this concern (Güneş et al., 2019; Sachdev et al., 2021). Total phenolic compounds and antioxidant activity have beneficial functions in plant protection. TPCs have hydroxyl groups which play significant roles against ROS. Therefore, TPCs in plants can be immediately matched to their AOA (Tosun et al., 2009). Furthermore, it has been found that phenols have antioxidant characteristics and have a distinct role in the system of enzyme activity and essential metabolic production (Jokar and Ronaghi, 2015). Moreover, there is an efficient role between phenolic compounds and antioxidant activity, which is good for plants according to studies on sunflower (Kiarostami et al., 2010), rosemary (Rady et al., 2011), lavender (Chrysargyris et al., 2018), and sweet basil (Ahmed et al., 2019). More studies discovered that various doses of N, P, and K affect the secondary metabolite production in plants (Stefanelli et al., 2011; Ibrahim et al., 2012). Similarly, application of K_2_SO_4_ as a foliar spray on Bouhouli trees led to an actual change in total phenolic and flavonoid content and an increase in chlorogenic acid relative to the control (Gaaliche et al., 2019). As verified by Jiang et al. (2024), the production of PAL and POD in the flowers of *Chrysanthemum morifolium* was increased by the utilization of 330 kg N + 196 kg P_2_O_2_ + 450 kg K_2_O per ha and 330 kg N + 196 kg P_2_O_5_ + 300 kg K_2_O per ha, respectively, in comparison with the control. Thus, 100% NPK significantly improved the chemical and biochemical contents in *C. erectus*, consequently resulting in good growth and aesthetic view for landscaping purposes.

### 4.2 Impact of natural extracts on vegetative, chemical, and biochemical traits

Our results suggested positive effects of the natural extracts used, especially ADY, on the studied parameters compared with the respective controls. The exogenous or foliar utilization of ADY, GT, and SW extracts either separately or together is an effective method because these extracts can be easily taken up by the leaves of plants and then translocated to the other plant organs. This leads to regulating and enhancing the metabolic processes in plant cells and decreasing the undesirable habitat effects. The literature highlighted the useful impacts of natural extracts on the various processes in cells of plants as ADY which increased the proline content and antioxidant enzymes and decreased the oxidative stress (Esmaeilzadeh Bahabadi and Sharifi, 2013) or decreased the lipid peroxidation (Alzandi and Naguib, 2022). Moreover, using ADY boosted the leaf N, P, K, TC, RWC, antioxidant enzymes (PPO, POD, and CAT), TPCs, and AOA, subsequently improving the growth traits via its contents of macro and micro nutrients, hormones, vitamins, proteins, carbohydrate, amino acids, and lipids (Amer, 2004; Ghoname et al., 2009). Furthermore, ADY extract has a useful role in vegetative growth and productivity stages with enhanced flower formation in some plants, which refers to its high auxin and cytokinin levels. It also improves the accumulation of carbohydrates (Sayed et al., 2019). Furthermore, they added that ADY extract has stimulatory impacts on cell division and elongation, protein and nucleic acid synthesis, and formation of chlorophyll. According to Khudair and Hajam (2021), ADY as foliar spray at 2 g L^-1^ enhanced the vegetative growth and root traits of Chinese carnation. They added that the combination between ADY at 2 mL L^-1^ and organic fertilizer (Algidex) at 2 or 4 mL L^-1^ increased the outmost vegetative, flower, and root traits of Chinese carnation. In a study of El-Shawa et al. (2020) on calendula plants, they concluded that foliar application with ADY at 4, 8, and 12 g L^-1^ caused increases in vegetative and flower traits, leaf contents of total chlorophyll, percentages of N, P, K, and RWC, and enzyme activity (PPO, POD, CAT, and SOD) in comparison with the control plants. Furthermore, they found that such levels of ADY reduced EL relative to the control plants of calendula. Moreover, ADY at 12 g L^-1^ positively enhanced the vegetative traits, total chlorophyll, carbohydrates, phenols, sugar, activities of PPO, CAT, and SOD of cowpea. The results demonstrated as well that the combination of 24-epibrassinolide (10 μM) and ADY (12 g L^-1^) was the best among the other combinations of such materials in promoting seed yield, POD weight, and the chlorophyll content of cowpea (Gholami et al., 2023). Most importantly, several studies have demonstrated the beneficial influences of seaweed extract on the morphological characteristics of plants and their chemical and biochemical contents, with subsequent encouragement in all physiological, and biochemical processes in plant cells by the contents of SW from different essential nutrients, hormones, amino acids, and polysaccharides (Craigie, 2011; Reddy and Saravanan, 2013; Jafarlou Bahmani et al., 2021). At low levels, SW extracts can cause various physiological plant responses, including improved plant growth, enhanced blooming and production, and elevated mineral content (Colla et al., 2017; Arioli et al., 2021). The improvement effect of SW extracts on the vegetative, chemical, and biochemical traits of *C. erectus* was compared to the control. This was due to SW composition that, like the natural growth hormones (auxins and cytokinins), enhances plant growth by raising the number of metabolic events (cell division and enlargement), in turn causing an increase in vegetative aspects (Prasad et al., 2010). Additionally, SW extract has a marked amount of macronutrients and micronutrients that have an essential role in the activation of many enzymes and coenzymes, which are employed in various biological processes, causing cell division and enlargement (Rokayya and Khojah, 2016). Additionally, algae have been applied to improve plant performance and tolerance to abiotic and biotic stresses due to their useful bio-stimulant content (Sharma et al., 2014).

Numerous studies on rose plants (Kularathne et al., 2021), *Lantana camara* and *Abelia x grandiflora* shrubs (Loconsole et al., 2022), *Citrullus lanatus* (Radwan et al., 2023), Kiwifruit (Rana et al., 2023), and strawberry (Bagh et al., 2024), have reported that the plants subjected to SW extracts as foliar application had a boost in growth traits and chemical and biochemical contents. Mohamed et al. (2021) concluded that treating sweet pepper with 3 g L^-1^ of ADY (a soil addition) combined with 3 g L^-1^ SW extract (as a foliar application) executed plausible results in terms of plant height, leaf area, and fresh and dry weight. The highest fruit values of carbohydrate, P, and K were achieved from the addition of folic acid plus seaweed extract at 3 g L^-1^. They added that the use of effective microorganisms and extracts of ADY and SW could lead to an increase in growth, element contents, activity of enzymes, fruit yield, and quality and nutritional value of sweet pepper fruit and also reduction of environmental contamination. According to uz Zaman et al. (2025), the utilization of SW extract at 1 or 3 g L^-1^ led to an increment in growth and biomass of hemp relative to the untreated control. The synergistic method of SW also effectively decreased H_2_O_2_ production, the accumulation of proline, and malondialdehyde generation but boosted the content of soluble sugars and protein relative to the control. They added that the cumulative effect of Cu stress can be avoidable with the application of the combination of SW extract + biochar + arbuscular mycorrhizal fungi, which resulted in increases in growth biomass, activity of antioxidant enzymes, and osmoprotectant contents via mitigating the accumulation of Cu. Furthermore, there are no studies on the effect of GT on the growth, chemical and biochemical contents, and physiological processes of *C. erectus* cells. This study has focused on the role of GT as a natural extract in the growth of plants and its attributes. The enhancement in the studied traits of *C. erectus* due to the application of GT may be attributed to its contents (tannins, volatile oil, caffeine, and the active polyphenol) which have stimulant effects. Moreover, its contents are from nutrients and vitamins, and these boost enzyme activity (Armstrong et al., 2020). Additionally, antioxidants in GT (catechin and tocophenol) have a distinct role in the maintenance of cells against ROS, which leads to the destruction of proteins, lipids, and DNA, thereby keeping the cells from aging and stimulating the vegetative growth of plants (Pastoriza et al, 2017; Ali et al., 2022).

Our results corresponded with those of Ahmed et al. (2014) who found that various levels of GT significantly boosted the leaves’ dry weight and the leaf area of Keitte mango. The 50 mg L^-1^ GT leaf extract promoted the seedling growth of *Allium cepa* under salt stress (Çavuşoğlu, 2020). Abdel Aal et al. (2021) showed that GT extract at 0.05%–2% had a favorable impact on all of the growth traits and nutritional levels of Barhee date plants. Furthermore, our results were supported by the results of Moustafa (2020) on aralia plants; he found that using GT extract at 0.1%–0.8% as foliar spray caused increments in plant height, number of leaves/plant, stem diameter, leaf area, number of branches/plant, fresh and dry weight of leaves, stems, and roots/plant, as well as leaf N%, P%, and K% and chl. a, b, and total chl. relative to the untreated plants. The study of Hassan and Ibrahim (2024) on the variety of caster (Zibo and Camencita) revealed that 75 mL L^-1^ GT extract achieved the highest dry weight of leaves and number of fruits/plant. Our results indicated that applying 3 g L^-1^ ADY is superior than the treatments using other natural extracts in enhancing the studied traits of *C. erectus* and may be due to 3 g L^-1^ ADY having more macronutrients and micronutrients, natural growth hormones, amino acids, proteins, lipids, polychloride compounds, enzymes, and carbohydrates. All of these materials lead to improved cell division and enlargement, increased plant nutrient contents, enhanced photosynthesis, carbohydrate accumulation, and enzyme activities as well as alleviating the harmful impacts of ROS. All of these lead to improved plant growth and development.

### 4.3 Impact of the interaction between NPK doses and natural extracts used on vegetative, chemical, and biochemical treats

The different combinations among NPK levels and natural extracts used caused considerable increases in the studied parameters over the application of NPK doses alone. Moreover, the combined 100% NPK dose with 3 g L^-1^ ADY was the best for the given high values of vegetative traits, chlorophyll index, RWC, enzyme activity (except CAT and PPO), TPCs, and AOA of *C. erectus*. These findings indicate that 100% NPK plus 3 g L^-1^ ADY have a significant amount of macronutrients and micronutrients in addition to the contents of ADY from stimulant compounds mentioned before. Our findings are consistent with those reported by Teja et al. (2017) in their study on annual chrysanthemum, where N and K were identified as essential components of chlorophyll, proteins, and amino acids. They noted that increased applications of N and K can enhance chlorophyll content, promote photosynthesis, and subsequently improve plant growth. Similarly, Nofal et al. (2021) observed the greatest enhancement in the leaf chemical composition of *Tagetes erecta* var. dwarf chrysanthemum—including the levels of N, P, K, total carbohydrates, chlorophyll a and b, total phenols, total indoles, and carotenoids—when plants were treated with 2 g pot^-1^ of NPK combined with SW extract at a concentration of 2 g L^-1^.

Previously, Amer et al. (2019) reported that treating sage plants with 75% NPK of the recommended dose combined with biofertilizer (*Azotobacter chroococcum*, *Bacillus megaterium* var. *phosphaticum*, and *B. cereus*) achieved the highest significant results of plant height, number of branches, fresh and dry weight, carbohydrate, chlorophylls, total phenol compounds, and antioxidants. Additionally, the application of yeast at 8 g L^-1^ plus chitosan at 300 mM caused an increase in plant height, RWC, chlorophyll a and b content, regulated antioxidant enzyme activities, and reduced oxidative stress signs in stressed garlic plants (Abdelaal et al., 2021). Previously, El-Azzony et al. (2018) documented that 75% NPK of the recommended dose plus effective microorganisms at 100 mL^-1^ per shrub was the best positive application to improve the vegetative growth of *Jatropha curcas.* Furthermore, Al-Hamzawi (2019) found that the combinations of seaweed and mixture of micronutrients at 1 and 2 mL L^-1^ significantly enhanced the vegetative and flowering traits of *Dianthus chinensis* and *Gazania* sp*lender*. Faizah et al. (2025) found that 10 g/plant NPK combined with 12 mL L^-1^ liquid organic fertilizer resulted in the best values of growth and yield parameters of the bean plant. In addition, BharathKumar et al. (2025), on *China aster* cv. “Azka × Kamini”, showed that treatment with 75% NPK dose + Azotobacter, PSB, and KSB biofertilizer + 0.5% SW extract reduced the lethal rate and increased the survival%, seedling height, and vigor indices. Thus, the combinations among NPK levels and natural extract (individually or together), especially 100% NPK with 3 g L^-1^ ADY, induced improvements in leaf chemical nutrients (N%, P%, and K%), chlorophyll index, RWC%, biochemical compounds (carbohydrate, activity of CAT, POD, and PPO, TPCs, and AOA) via sufficient amounts of essential elements of 100% NPK dose and natural extracts used. In particular, ADY plus the different biochemical compounds of ADY, SW, and GT mentioned previously account for the growth, development, and, consequently, good aesthetic characteristics of *C. erectus* for landscape purposes.

## 5 Conclusion

This study highlights the effectiveness of integrating chemical fertilizers (NPK) with natural extracts (ADY, GT, and SW) in enhancing the overall performance of *C. erectus*, particularly its chemical and biochemical attributes. The combined application of natural extracts and chemical fertilizers positively influences plant growth by promoting physiological, chemical, and biochemical processes as well as enhancing cell division and elongation, ultimately leading to improved growth characteristics. The application of 100% NPK, 3 g L^-1^ ADY, and their combination produced the most significant improvements in the vegetative, chemical, and biochemical traits of *C. erectus*. The addition of 3 g L^-1^ ADY can enhance nutrient uptake efficiency and potentially reduce the need for applying NPK fertilizers beyond the suggested 100% dose. Therefore, the integration of NPK with natural extracts, particularly ADY, can be utilized to enhance the aesthetic qualities of *C. erectus* for landscape applications while simultaneously reducing environmental contamination associated with excessive chemical fertilizer use. Further studies on the nutritional requirements of ornamental shrubs and trees are essential to develop optimal fertilization regimes tailored to each species.

## Data Availability

The raw data supporting the conclusions of this article will be made available by the authors, without undue reservation.
